# Hydro, wind and solar power as a base for a 100% renewable energy supply for South and Central America

**DOI:** 10.1371/journal.pone.0173820

**Published:** 2017-03-22

**Authors:** Larissa de Souza Noel Simas Barbosa, Dmitrii Bogdanov, Pasi Vainikka, Christian Breyer

**Affiliations:** 1 Luiz de Queiroz College of Agriculture, University of São Paulo, Piracicaba, São Paulo, Brazil; 2 VTT Technical Research Centre of Finland Ltd., Lappeenranta, Finland; 3 Lappeenranta University of Technology, Lappeenranta, Finland; Centro de Investigacion Cientifica y de Educacion Superior de Ensenada Division de Fisica Aplicada, MEXICO

## Abstract

Power systems for South and Central America based on 100% renewable energy (RE) in the year 2030 were calculated for the first time using an hourly resolved energy model. The region was subdivided into 15 sub-regions. Four different scenarios were considered: three according to different high voltage direct current (HVDC) transmission grid development levels (region, country, area-wide) and one integrated scenario that considers water desalination and industrial gas demand supplied by synthetic natural gas via power-to-gas (PtG). RE is not only able to cover 1813 TWh of estimated electricity demand of the area in 2030 but also able to generate the electricity needed to fulfil 3.9 billion m^3^ of water desalination and 640 TWh_LHV_ of synthetic natural gas demand. Existing hydro dams can be used as virtual batteries for solar and wind electricity storage, diminishing the role of storage technologies. The results for total levelized cost of electricity (LCOE) are decreased from 62 €/MWh for a highly decentralized to 56 €/MWh for a highly centralized grid scenario (currency value of the year 2015). For the integrated scenario, the levelized cost of gas (LCOG) and the levelized cost of water (LCOW) are 95 €/MWh_LHV_ and 0.91 €/m^3^, respectively. A reduction of 8% in total cost and 5% in electricity generation was achieved when integrating desalination and power-to-gas into the system.

## Introduction

South and Central America are economically emerging regions that have had sustained economic growth and social development during the last decade. The regions’ 3% gross domestic product (GDP) growth rate [1] followed by an estimated fast-paced electricity demand growth over the coming decades [2] requires the development of the power sector in order to guarantee efficiency and security of supply.

The South and Central American electrical energy mix is the least carbon-intensive in the world due to the highest share of renewable energy, mainly based on hydropower installed capacities [3, 4]. However, the need to reduce the vulnerability of the electricity system to a changing hydrological regime is evident. Natural climate variability and climate change have been modifying the hydrological cycle and water regime in the drainage basis, threatening the availability and reliability of hydropower sources of many countries in the region, especially Brazil [5]. Serious droughts and severe weather events in Brazil have caused a reduction of 45% in the average water levels in hydro dam reservoirs in the last four years [6], and due to the fact that 71% of the electricity supply in the country relies on hydropower [7], the changes have endangered the country’s electricity security and supply. Over the past decade hydropower’s share in South and Central America has been declining and the indications for the future are that the downward trend will continue [2]. Regarding non-hydro renewable energy (RE) potential, South and Central America have vast solar, wind and biomass potentials, which could allow the region to maintain its high share of renewables, even under a low hydropower future scenario [2].

Most parts of the region lies within the Sun Belt region of highest solar radiation [8], with Chile, Bolivia and Argentina among the ten countries in the world with maximum irradiation for fixed, optimally tilted PV systems [9]. Moreover, the Atacama Desert has the best global maximum solar irradiation of 2,770 kWh/(m^2^∙a) (for fixed, optimally tilted PV systems) and is an excellent region for solar photovoltaics (PV) energy production [9].

Regarding the potential for wind energy generation, Brazil (northeast region), Chile (northwest region), Paraguay (north region), Bolivia (southeast region) and Argentina (south and east region) have high annual wind energy potentials [10], which make the region highly valuable for wind power. In fact, one of the best wind sites globally is located in the region of Patagonia, Argentina.

Concerning biomass resources, South and Central America have suitable climatic conditions, land availability and cheap labor when compared to other countries [11]. In total biofuel production, Brazil and Argentina are, respectively, the second biggest ethanol and biodiesel producers globally and a recent wave of investments from the governments has boosted the production of biofuels over the medium and long terms [11, 12]. In addition, South and Central American solid wastes, and agricultural and industrial residues are able to generate 1025 TWh_LHV_ per year in the region [13].

Added to the above mentioned facts, a few numbers of South American countries have been supported not only by a regulatory framework that has raised investments in renewable energy generation, but also by low-carbon development plans. Long-term electricity auctions, aiming either at guaranteeing the adequacy of the system or at RE system electricity support, have been occurring in South and Central American countries [14]. Over 13,000 MW of capacity has been contracted through tendering since 2007 in Argentina, Brazil, Chile and Peru [15]. Competitive bidding in Uruguay has reached the country target of 1 GW of wind power capacity by 2015 and Central American countries such as El Salvador, Guatemala, Honduras and Panama released bids for renewable energy in 2014 [15]. Brazil, Colombia, Bolivia, Chile, Costa Rica and Peru have national plans with climate change mitigation initiatives and scenarios [16, 17] that can lead to national sustainable development and drive the changes in the countries’ energy systems. Chile’s government roadmap, launched in September 2015, is an excellent example of initiative since the report calls for no less than 70% of the country’s electricity demand being met by renewable energy sources by 2050, with an increase in 58% of actual renewable energy sources [18]. Costa Rica had been very close to reaching the 100% RE target already in 2015, since for 94 consecutive days of the year the total electricity had been covered by RE and the country reached 98% in total for the year [19]. Uruguay has slashed its carbon footprint in the last 10 years and, despite already having 94.5% of its electricity and 55% of its overall energy mix provided by RE, has announced a 88% cut in carbon emissions by 2017 compared with the average for 2009–13 [20].

As long as the energy systems in the region have a broad range of possible RE options and solutions supported by a regulatory framework, it has an essential role in addressing climate change and limiting global warming to less than 2°C compared to pre-industrial levels. High shares of renewables for the Latin American energy system have been outlined in other modelling studies for the year 2050, such as in [21] and [17]. Martínez et al. (2015) have considered three different assessment models to determine the energy and emissions trends in Brazil and the rest of the Latin American region up to 2050 based on a set of scenarios consistent with current trends and with the 2°C global mitigation target [17]. Greenpeace (2015) reports a compelling vision of what an energy future may look like for a sustainable world [21]. It presents two global scenarios in which energy is supplied 100% by renewable energy technologies with different reductions on energy intensity. The main differences between these studies and the study presented in this paper concern methodology and the existence of flexibility options for an overall balanced system. Both Martínez et al. (2015) and Greenpeace (2015) studies [21, 17], for instance, have considered yearly resolution models (and not hourly resolution models) for RE generation, energy demand and supply. This approach, however, is not appropriate for systems relying on high shares of renewable energy since the energy generation varies hourly over time and does not guarantee that the hourly energy supply in a year covers the local demand from all sectors. Furthermore, the existence of different types of flexibility in the system, such as demand side management and energy shifted in location (transmission grids connecting different locations) were not evaluated in these studies either, and storage of energy at one location (and energy shifted in time) was only mentioned and not quantified.

Other studies [22, 23, 24, 25, 26] have performed the optimization of energy systems on an hourly basis with a high penetration of renewable energy for countries such as Ireland, USA, Australia and Northeast Asia. This study, using a similar hourly based model and analysing different grid development levels, aims at designing an optimal and cost competitive 100% RE power system for South and Central America. A potential evolution of the generation mix was considered and takes into account:

- the actual electricity trade and transmission infrastructure of different sub-regions of South and Central America
- an optimal system design and wise utilization of considered available RE resources
- synergy between various resources and different regions that increase the efficiency of the power sector

Three different scenarios with different high voltage direct current (HVDC) transmission grid development levels (region-wide, country-wide and area-wide energy systems) and one integrated scenario were analysed and compared. The integrated scenario considers an additional electricity demand for water desalination and industrial natural gas production, in order to give the system flexibility and to decrease overall cost guarantee that the water demand of the region will be fulfilled.

## Methodology

The energy system model used in this study is based on linear optimization of energy system parameters under applied constraints and is composed of a set of power generation and storage technologies, as well as water desalination and synthetic natural gas (SNG) generation via power-to-gas (PtG) for industrial use, which operate as flexible demand. For a complete understanding of the whole energy system, a fully integrated scenario that also considers heat and mobility demand has to be modeled, even though this is not in the scope of this study. As the applied energy system model has already been described in [23] and [27], the coming sections do not include a detailed description of the model, its input data and the applied technologies. However, it presents a comprehensive summary and all additional information that has been assumed for the model in the present study. Further technical and financial assumptions can be found in the Supporting Information section in this paper.

### Model summary

The energy system optimization model is based on a linear optimization of the system parameters under a set of applied constraints as described in detail previously [23 and 27]. The main constraint for the optimization is to guarantee that for every hour of the year the total electric energy supply within a sub-region covers the local demand from all considered sectors and enables a precise system description including synergy effects of different system components for the power system balance.

The aim of the system optimization is to achieve a minimal total annual energy system cost. The annual energy system cost can be calculated as the sum of the annual costs of installed capacities of the different technologies, costs of energy generation and costs of generation ramping. On the other hand, for residential, commercial and industrial electricity prosumers the target function is minimal cost of consumed energy, calculated as the sum of self-generation, annual cost and cost of electricity consumed from the grid, minus benefits from selling of excess energy. Prosumers are the ones that install respective capacities of rooftop PV systems and batteries and produce and consume electricity at the same time.

**Fig 1:**
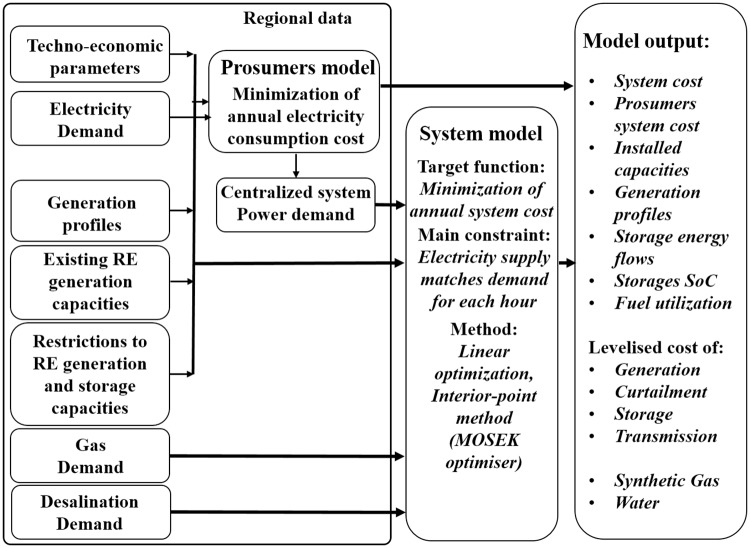
Model flow diagram.

The model flow diagram that contains all the considered input data, system models and model output data is presented on Fig 1.

### Input data

Several types of input datasets and constraints are used in the model, as described previously by [23] and [27]:

- historical weather data for direct and diffuse solar irradiation, wind speed, precipitation amounts and geothermal data,
- synthetic load data for every sub-region,
- water and industrial natural gas demand,
- technical characteristics of used energy generation, storage and transmission technologies, such as power yield, energy conversion efficiency, power losses in transmission lines and storage round trip efficiency,
- capital expenditures, operational expenditures and ramping costs for all technologies,
- electricity costs for residential, commercial and industrial prosumers,
- limits for minimum and maximum installed capacity for all energy technologies,
- configuration of regions and interconnections.

Description of historical weather data can be found in [23] and [27] and is not highlighted in this paper.

Geothermal data are evaluated based on existing information on the surface heat flow rate [28, 29] and surface ambient temperature for the year 2005 globally. For areas where surface heat flow data are not available, an extrapolation of existing heat flow data was performed. Based on that, temperature levels and available heat of the middle depth point of each 1 km thick layer, between depths of 1 km and 10 km [30, 31, 32] globally with 0.45°x0.45° spatial resolution, are derived.

Due to the fact that in the future, depletion and deterioration of available water resources can lead to water shortages, water demand was calculated based on water consumption projections and future water stress [33]. Water stress occurs when the water demand exceeds renewable water availability during a certain period of time. It is assumed that water stress greater than 50% shall be covered by seawater desalination and that there are no restrictions on the variable operation of the desalination plants [34, 35]. Transportation costs are also taken into account and the methodology and calculations for seawater desalination are described in [36]. The energy consumption of seawater reverse osmosis desalination plants is set to 3.0 kWh/m^3^ and horizontal and vertical pumping are 0.04 kWh/(m^3^∙h∙100km) and 0.36 kWh/(m^3^∙h∙100m), respectively [36]. The levelized cost of water (LCOW) includes water production, electricity, water transportation and water storage costs and will change according to renewable resource availability and cost of water transport to demand sites.

Present industrial gas consumption is based on natural gas demand data from the International Energy Agency statistics [37] and natural gas consumption projections for the year 2030 were calculated considering industrial annual growth projections based on the World Energy Outlook [1].

### Applied technologies

The technologies used in the South and Central American energy system optimization can be divided into four different categories: conversion of RE resources into electricity, energy storage, energy sector bridging (for definition, see later), and electricity transmission.

The RE technologies for producing electricity applied in the model are ground-mounted (optimally tilted and single-axis north-south oriented horizontal continuous tracking) and rooftop solar PV systems, concentrating solar thermal power (CSP), onshore wind turbines, hydropower (run-of-river and dams), biomass plants (solid biomass and biogas), waste-to-energy power plants and geothermal power plants. Hydro run-of-river plants are the ones located in rivers that have a small reservoir capacity that stores maximum 48 full load hours of water in energy and hydro dams are the ones with bigger reservoirs, capable of storing energy up to months.

For energy storage, batteries, pumped hydro storage (PHS), adiabatic compressed air energy storage (A-CAES), thermal energy storage (TES) and power-to-gas (PtG) technology are integrated to the energy system. PtG includes synthetic natural gas (SNG) synthesis technologies: water electrolysis, methanation, CO_2_ scrubbing from air, gas storage, and both combined and open cycle gas turbines (CCGT, OCGT). The synchronization of the operation of SNG synthesis technologies are important once the model does not include hydrogen and CO_2_ storage. A 48-hour biogas buffer storage allows part of the biogas to be upgraded to biomethane and injected into the gas storage.

The energy sector bridging technologies provide more flexibility to the entire energy system, thus reducing the overall cost. One bridging technology available in the model is PtG technology for the case that the produced gas is consumed in the industrial sector and not as a storage option for the electricity sector. The second bridging technology is seawater reverse osmosis (SWRO) desalination, which couples the water sector to the electricity sector.

For electricity transmission most transmission lines are based on high voltage alternating current (HVAC) technology. However, for better efficiency over very long distances high voltage direct current (HVDC) technology are usually used. Alternating current (AC) grids within the sub-regions exist but are beyond of the methodological options of the current model since grid costs and distribution data are not accessible for the entire region and, therefore, would implicate a bad estimation on the respective costs and grid distribution. However, for inter-regional electricity transmission, HVDC grids are modeled. Power losses in the HVDC grids consist of two major components: length dependent electricity losses of the power lines and losses in the converter stations at the interconnection with the AC grid.

An energy system mainly based on RE and in particular intermittent solar PV and wind energy requires different types of flexibility for an overall balanced and cost optimized energy mix. The four major categories are generation management (e.g. hydro dams or biomass plants), demand side management (e.g. PtG, SWRO desalination), storage of energy at one location and energy shifted in time (e.g. batteries), and transmission grids connecting different locations and energy shifted in location (e.g. HVDC transmission).

**Fig 2:**
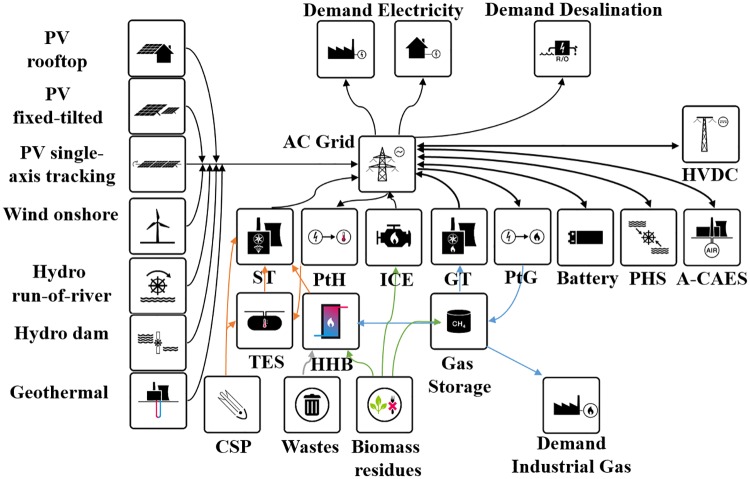
Block diagram of the energy system model for South and Central America.

The full model block diagram is presented in Fig 2.

### Scenario assumptions

#### Regions subdivision and grid structure

The South America region and also Central American countries that connect South America to North America (Panama, Costa Rica, Nicaragua, Honduras, El Salvador, Guatemala and Belize) were considered in this study. The super region was divided into 15 sub-regions: Central America (that accounts for Panama, Costa Rica, Nicaragua, Honduras, El Salvador, Guatemala and Belize), Colombia, Venezuela (that accounts for Venezuela, Guyana, French Guiana, Suriname), Ecuador, Peru, Central South America (that accounts for Bolivia and Paraguay), Brazil South, Brazil São Paulo, Brazil Southeast, Brazil North, Brazil Northeast, Argentina Northeast (includes Uruguay), Argentina East, Argentina West and Chile. Brazil and Argentina, which are the biggest countries in population and territory, were divided into five and three sub-regions respectively, according to area, population and national grid connections.

In this paper four scenarios for energy system development options are discussed:

- regional energy systems, in which all the regions are independent (no HVDC grid interconnections) and the electricity demand has to be covered by the respective region’s own generation;
- country-wide energy system, in which the regional energy systems are interconnected by HVDC grids within the borders of nations;
- area-wide energy system, in which the country-based energy systems are interconnected;
- integrated scenario: area-wide energy system scenario with SWRO desalination and industrial natural gas demand. In this scenario, RE sources combined with PtG technology are used not only as electricity generation and storage options within the system, but also as energy sector bridging technologies to cover water desalination and industrial gas demand, increasing the flexibility of the system.

**Fig 3:**
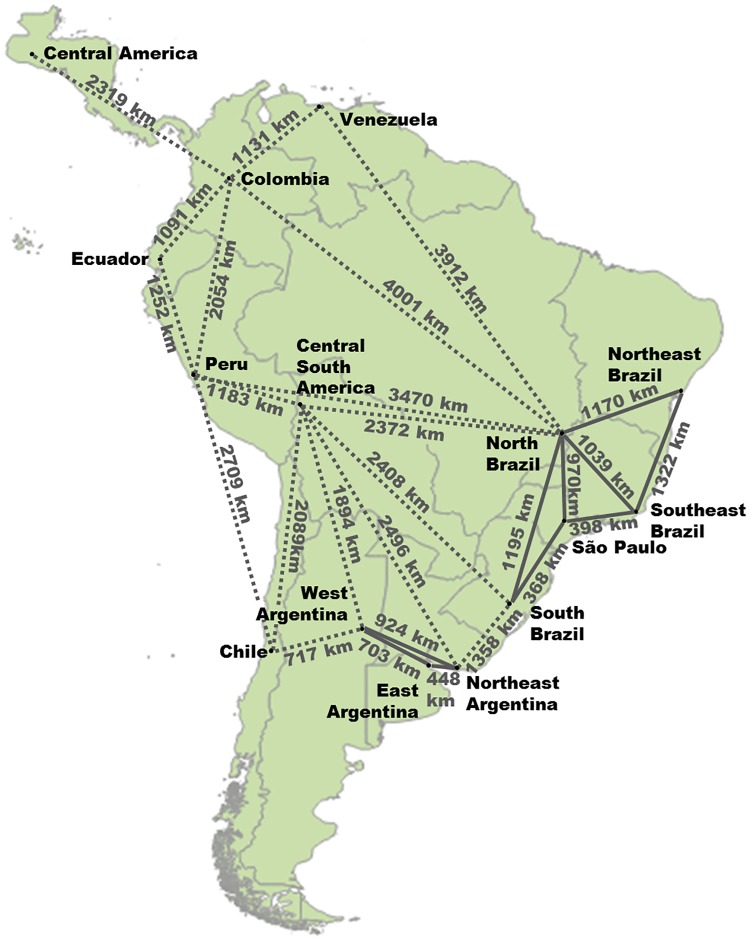
South and Central American sub-regions and HVDC transmission lines configuration.

Fig 3 presents the South and Central American region’s subdivision and grid configuration. HVDC interconnections for energy systems of the countries are shown by dashed lines. The structure of HVDC grid is based on existing configuration of South and Central American grids.

#### Financial and technical assumptions

The model optimization is performed in a technological and financial status for the year 2030 in a currency value of the year 2015. The overnight building approach as typically applied for nuclear energy [38] was considered. The financial assumptions for capital expenditures (capex), operational expenditures (opex) and lifetimes of all components, for all the considered scenarios, are provided in Table A in S1 File. Weighted average cost of capital (WACC) is set to 7% for all scenarios, but for residential PV self-consumption WACC is set to 4%, due to lower financial return requirements. The technical assumptions concerning power to energy ratios for storage technologies, efficiency numbers for generation and storage technologies, and power losses in HVDC power lines and converters are provided in Tables A, B and C in S1 File. Since the model calculates electricity generated by prosumers, electricity prices for residential, commercial and industrial consumers in most of the region countries for the year 2030 are needed, being taken from [39] except for Ecuador, Suriname, Venezuela, Guyana and French Guiana, whose electricity prices are taken from local sources. Prices are provided in Table E in S1 File. As the electricity price is on a country basis, the sub-regions’ electricity prices in Brazil and Argentina have the same value. The production and consumption of electricity by prosumers are not simultaneous and, consequently, prosumers cannot self-consume all electricity generated by their solar PV system. The excess electricity produced by prosumers is assumed to be fed into the grid for a transfer selling price of 2 €cents/kWh. Prosumers cannot sell to the grid more power than their own annual consumption.

#### Feed-in profiles for solar and wind energy

**Fig 4:**
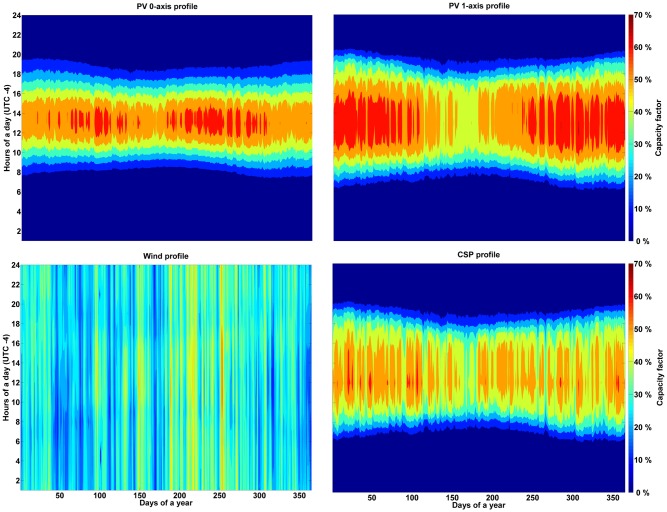
Aggregated feed-in profiles for optimally tilted PV (top left), single-axis tracking PV (top right), 3 MW 150 m hub height wind turbine (bottom left) and CSP solar field (bottom right).

The feed-in profiles for solar CSP, optimally tilted and single-axis tracking PV, and wind energy were calculated according to [23] and [27]. Fig 4 presents the aggregated profiles of solar PV generation (optimally tilted and single-axis tracking), wind energy power generation and CSP solar field. The profiles are normalized to maximum capacity averaged for South America. A table with the computed average full load hours (FLH) is provided in Table F in S1 File.

The feed-in values for hydropower are calculated based on the monthly resolved precipitation data for the year 2005 as a normalized sum of precipitation in the regions. Such an estimate leads to a good approximation of the annual generation of hydropower plants, as described previously in [23].

#### Biomass and geothermal heat potentials

For biomass and waste resource potentials, data is taken from [13] and classified as described in [23]. Costs for biomass are calculated using data from the International Energy Agency [40] and Intergovernmental Panel on Climate Change [41]. For solid wastes a 75 €/ton gate fee for incineration is assumed. Calculated solid biomass, biogas, solid waste and geothermal heat potentials and prices for biomass fuels are provided in Tables G and H in S1 File. Price differences between countries are because of different waste and residue component shares. Heating values are based on lower heating values (LHV).

For regional geothermal heat potentials the calculations are based on spatial data for available heat, temperature and geothermal plants for depths from 1 km to 10 km. Geothermal heat is used only for electricity generation in the model. For each 0.45°x0.45° area and depth, geothermal LCOE is calculated and optimal well depth is determined. It is assumed that only 25% of available heat will be utilized as an upper resource limit. The total available heat for the region is calculated using the same weighed average formula as for solar and wind feed-in explained in [23], except for the fact that areas with geothermal LCOE exceeding 100 €/MWh are excluded.

#### Upper and lower limitations on installed capacities

Lower and upper limits calculations are described in [23]. Lower limits on already installed capacities in South and Central American sub-regions are provided in Table I in S1 File and all upper limits of installable capacities in South and Central American sub-regions are summarized in Table J in S1 File. For other technologies, upper limits are not specified unless for biomass residues, biogas and waste, for which it is assumed that the available and specified amount of the fuel can be used during the year.

#### Load

**Fig 5:**
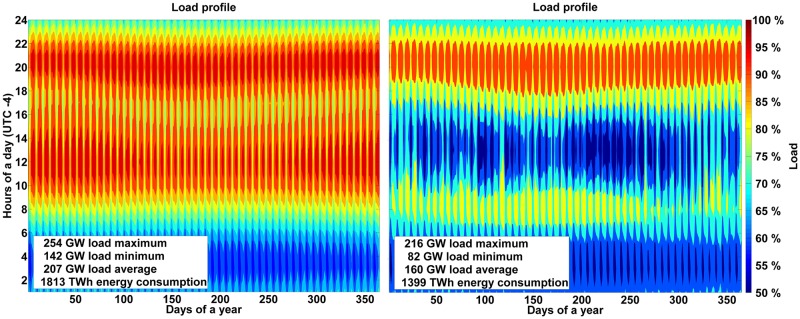
Aggregated load curve (left) and system load curve with prosumers influence (right) for the year 2030.

The demand profiles for sub-regions are calculated using a synthetic algorithm, calibrated according to previous load curves for Argentina, Brazil and Chile [42]. The data is in hourly resolution for the year 2015. It is computed as a fraction of the total country energy demand based on load data weighted by the sub-regions’ population. Fig 5 represents the area-aggregated demand of all sub-regions in South and Central America. The increase in electricity demand by year 2030 is estimated using IEA data [1] and local data. Solar PV self-consumption prosumers have a significant impact on the residual load demand in the energy system as depicted in Fig 5 (right). The overall electricity demand and the peak load are reduced by 22.8% and 15.0%, respectively, due to prosumers.

Industrial gas demand values (gas demand excluding electricity generation and residential sectors) and desalinated water demand for South and Central American sub-regions are presented in Table K in S1 File. Gas demand values are taken from IEA data [37] and desalination demand numbers are based on water stress and water consumption projection [36].

## Results

### Main findings on the optimized energy system structure and costs

As the main results, cost minimized electrical energy system configurations are derived for the given constraints for all the studied scenarios. The configurations are also characterized by optimized installed capacities of RE electricity generation, storage and transmission for every modelled technology and hourly electricity generation, storage charging and discharging, electricity export, import, and curtailment are calculated. In order to determine whether or not the project is interesting compared to other similar project’s average rates, the average financial results of the different scenarios for the total system (including PV self-consumption and the centralized system) are expressed as levelized costs. The levelized costs used are: levelized cost of electricity (LCOE), levelized cost of electricity for primary generation (LCOE primary), levelized cost of curtailment (LCOC), levelized cost of storage (LCOS) and levelized cost of transmission (LCOT). All levelized costs, total annualized cost, total capital expenditures, total renewables capacity and total primary generation for South and Central America region are presented in Table 1.

**Table 1:** Financial results for the four scenarios applied in South and Central America regions.

	Total LCOE	LCOE primary	LCOC	LCOS	LCOT	Total ann. cost	Total CAPEX	RE capacities	Generated electricity
	[€/MWh_el_]	[€/MWh_el_]	[€/MWh_el_]	[€/MWh_el_]	[€/MWh_el_]	[b€]	[b€]	[GW]	[TWh_el_]
Region-wide	61.9	41.6	3.0	17.3	0	115	948	798	2080
Country-wide	59.1	40.2	2.2	15.6	1.1	109	912	744	1978
Area-wide	56.5	40.6	1.3	11.5	3.1	104	889	690	1905
Integration scenario	46.6	36.3	1.0	7.6	1.7	153	1354	1173	2970

In Table 1 the importance of HVDC transmission lines in 100% RE systems is clear: it leads to a significant reduction in RE installed capacities, electricity cost, annual expenditures for the system and storage costs; electricity cost of the entire system in the case of area-wide open trade power transmission decreases by 4.4% and 8.7% compared to the country-wide and region-wide scenarios, respectively. Grid utilization decreases the primary energy installed conversion capacities by 7.3% and 13.5% in reference to country-wide and region-wide scenarios, respectively, and reduces storage utilization, according to Table 2. Cost of transmission is relatively small in comparison to the decrease in primary generation and storage costs. Curtailment costs are reduced by 40.9% and 56.7% in the area-wide scenario compared to the country-wide and region-wide scenarios, respectively, decreasing more significantly than storage costs in the case of broader grid utilization; however, the impact of excess energy on total cost is rather low.

**Table 2:** Overview on installed RE technologies and storage capacities for the four scenarios.

		Region-wide	Country-wide	Area-wide	Integration scenario
PV self-consumption	[GW]	267.8	267.8	267.8	267.8
PV optimally tilted	[GW]	1.2	1.2	1.2	1.2
PV single-axis tracking	[GW]	209.2	188.2	146.5	557.2
PV total	[GW]	478.2	457.2	415.5	826.2
CSP	[GW]	0	0	0.1	0
Wind energy	[GW]	83.4	68.7	68.6	134.4
Biogas power plants	[GW]	24.1	19.2	17.3	18.1
Biomass power plants	[GW]	8.1	2.9	2.8	2.6
MSW incinerator	[GW]	0.9	0.9	0.9	0.9
Geothermal energy	[GW]	0	0	0	0
Hydro Run-of-River	[GW]	39.7	39.7	38.7	38.7
Hydro dams	[GW]	143.6	142.8	144.3	144.0
Battery PV self-consumption	[GWh]	412.0	412.0	412.0	412.0
Battery total	[GWh]	661.8	654.1	538.1	607.2
PHS	[GWh]	1.2	1.1	1.1	1.1
A-CAES	[GWh]	122.6	0.2	8.7	0
Heat storage	[GWh]	0	0.1	3.4	0
PtG electrolyzers	[GW_el_]	10.1	7.2	0.5	131.4
CCGT	[GW]	32.6	31.0	23.4	5.4
OCGT	[GW]	6.0	5.7	4.3	4.9
Steam Turbine	[GW]	4.0	0	0.2	0

A further decrease in LCOE of 17.5% compared to the area-wide open trade scenario can be reached by the integration of water desalination and industrial gas sectors. This cost reduction is mainly explained by a reduction of storage cost by 35% since industrial gas and desalination sectors decrease the need for long-term storage utilization, giving additional flexibility to the system through demand management. An 11% decrease in primary electricity generation cost can be noticed as well and is explained by an increase in the flexibility of the system and the utilization of low-cost wind and solar electricity as can be seen in Table 2. For biogas, a fraction of 24% of the biogas used in biogas power plants in the area wide-open trade scenario is re-allocated from the electricity sector to the industrial gas demand for efficiency reasons. The sub-region Brazil Northeast has a peculiarity that has to be highlighted in the integrated scenario: 26.8 TWh of its industrial gas demand is supplied only by biogas plants and no PtG is needed (Table K in S1 File. The numeric values for LCOE components in all sub-regions and scenarios are summarized in Table N in S1 File.

Concerning RE installed capacities, all the RE technologies present a reduction of total installed capacity with an increase of grid utilization (Table 2); solar PV technologies have the highest GW installed capacity in all the analyzed scenarios, accounting for 61%, 62%, 60% and 71% of the total installed capacity in region-wide, country-wide, area-wide and integrated scenarios, respectively. The high share of solar PV can be explained by the fact that this is the least cost RE source for the region as a whole, as a consequence of assuming a fast cost reduction of solar PV and battery storage in the next fifteen years [43, 44]. Furthermore, the area-wide open trade scenario leads to 64% of solar PV total installed capacity being provided by PV prosumers as a result of prosumer LCOE competitiveness all over the region.

A PV self-consumption overview is given in Table L in S1 File. Self-generation plays a crucial role in 100% RE power systems for South and Central America due to rather high electricity prices throughout South and Central America and low self-consumption LCOE. Self-generation covers 99.3% of residential prosumers’ demand, 91.6% and 92% of demand for commercial and industrial prosumers.

Despite the fact that an upper limit 50% higher than the current capacity was considered for hydro dams and hydro RoR plants, the total hydropower plants’ installed capacity practically did not change considering all the studied scenarios: PV and wind seemed to be more profitable technologies according to the availability of the regions’ resources.

For energy storage options, transmission lines decrease the need for storage technologies, since energy shifted in time (storage) can be partly cost effectively substituted by energy shift in location; total installed capacities of batteries, PHS, A-CAES, PtG and gas turbines decrease with the grid expansion. PtG electrolyzers have a rather low installed capacity in the region-wide and country-wide scenarios and for the area-wide scenario, PtG is not needed for seasonal storage. On the other hand, hydro dams have a key role as virtual batteries for solar and wind long-term balancing, reducing interregional electricity trade and electricity transmission costs.

Concerning water desalination need, although the South American region has high water availability and rainfall, regions such as Chile, the western part of Argentina and Venezuela, shall present a need for water desalination by 2030 according to water stress calculations (Table K in S1 File.

**Fig 6:**
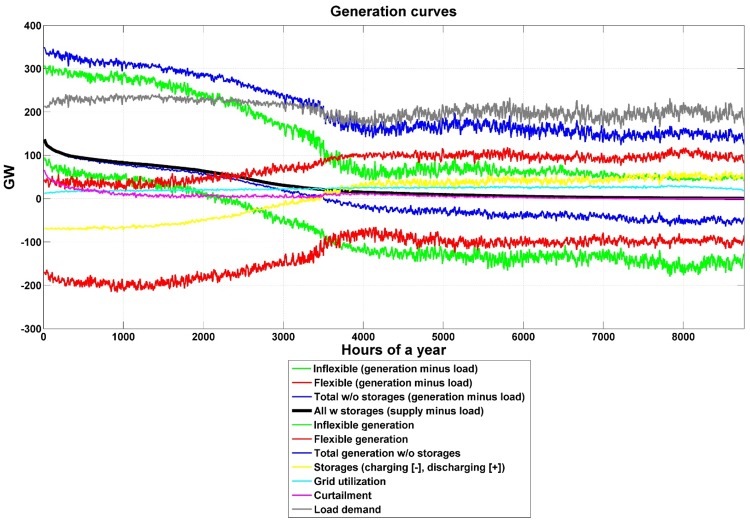
Electricity generation duration curves for the area-wide open trade scenario for South and Central America.

An overview of the electricity generation curves for the area-wide scenario can be seen in Fig 6. All 8760 hours of the year are sorted according to the generation minus the load, which is represented by the black line. A higher electricity generation than demand can be observed for 3500 hours of the year, which is used for charging storage. This is caused by a high electricity generation from inflexible energy sources, due to high shares of solar PV and wind energy in the South and Central American energy mix, and a higher solar irradiation and wind speed in the region during these hours of the year. As a consequence, flexible electricity generation options (such as hydro dams, biomass and biogas) and discharge of storage plants are needed. On the other hand, during the other hours of the year, the inflexible electricity generation reduces significantly in comparison to the decrease in electricity demand, increasing the need for flexible electricity generation, energy storage discharge and grid utilization. The storage plants are operated for about 3500 hours of the year in charging mode and about 5250 hours in discharging mode. Electricity curtailment is only significant for some hundreds of hours in the year and constant during almost the entire period since the existence of HVDC transmission lines enables that sub-regions with the best RE resources to export electricity to the ones with a shortage in RE resources.

### Main findings on the optimized energy system structure in a sub-region analysis

**Fig 7:**
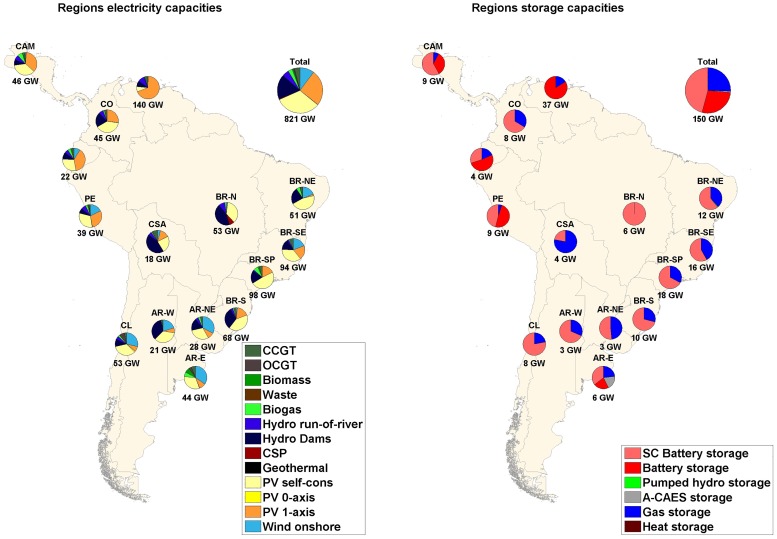
Installed capacities of RE generation (left) and storage technologies (right) for region-wide scenario.

**Fig 8:**
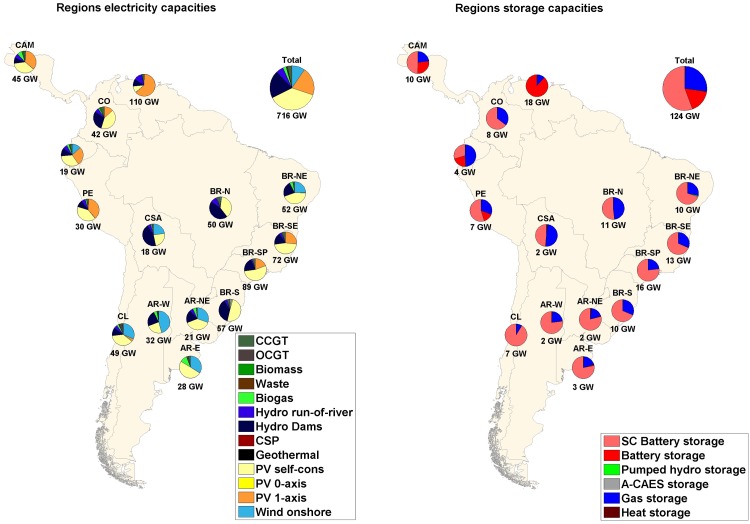
Installed capacities of RE generation (left) and storage technologies (right) for area-wide open trade scenario.

**Fig 9:**
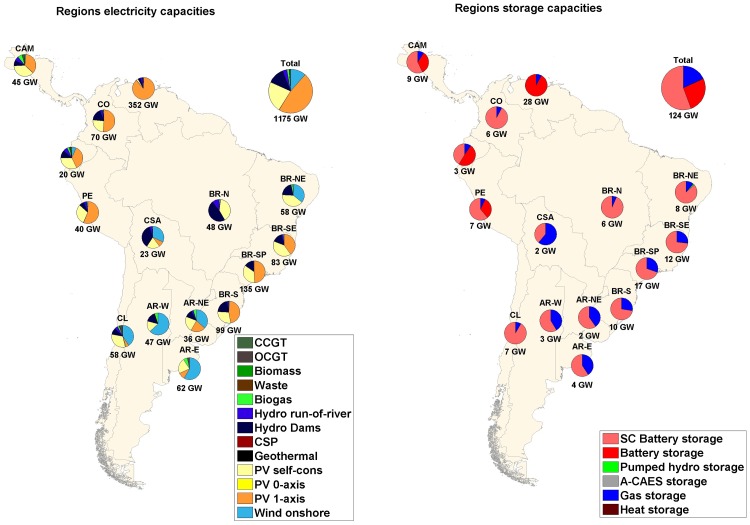
Installed capacities of RE generation (left) and storage technologies (right) for integrated scenario.

If a sub-regional analysis is considered, as presented in Figs 7–9, some differences between the scenarios, especially between the area-wide and the integrated scenarios, can be noticed. Additional demand in the case of a RE-based energy system can change the entire system structure because of shifting optimal cost structure parameters and areas being confronted with their upper resource limits. For region-wide and area-wide scenarios, solar PV dominates in almost all the sub-regions considered; for the integrated scenario, in which an additional electricity demand was included, the sub-regions that have excellent wind conditions and, therefore, low cost wind energy, have high shares of wind installed capacities in their energy mix. The shift to power in the industrial gas and desalination sectors is driven by a higher supply of least cost wind sites in sub-regions such as Central South America, Brazil Northeast, Argentina East, Argentina Northeast, Argentina West and Chile. Still considering the integrated scenario, for all other sub-regions, the increase in electricity demand system flexibility is followed by an increase in solar PV single-axis installed capacities, being in this case, the least cost RE source.

The interconnected HVDC transmission grid significantly decreases total installed capacities (Fig 7 and Table 2): mainly solar PV single-axis (i.e. PV single-axis installed capacities are reduced by 100% in Argentina East from region-wide to area-wide scenario) and wind turbines (i.e. wind installed capacities are decreased by 99.8% in Brazil Southeast from region-wide to area-wide scenario) for almost all the sub-regions. Some exceptions are Central South America, Brazil Northeast and Argentina West, that had an increase in 1.6%, 4.3% and 51.9%, respectively, in total RE installed capacities from region-wide to area-wide scenario. Despite a significant reduction in PV single-axis capacities (99.7%, 98.7%, 99.3%, respectively), an increase in wind capacities (486.7%, 34.8% and 244.4%, respectively) was observed due to excellent wind energy conditions in the respective sub-regions. The structure of HVDC power lines and utilized RE resources strongly influence the total storage capacity needed. In this context, the already installed hydro dams are an important RE source that can act as virtual batteries for long-term storage. Data of storage systems’ discharge capacities, energy throughput and full load cycles per year are summarized in Table M in S1 File. The generation capacities of storage technologies decrease with integration of the HVDC grid. However, for the integrated scenario capacities of storage technologies increase in absolute numbers. State-of-charge profiles for the area-wide scenario for battery, PHS, A-CAES and gas storages and hydro dams are provided in Fig E and F in S2 File. The state-of-charge diagrams show the system optimized operation mode of the different storage technologies: mainly daily (battery, PHS), mainly weekly (A-CAES) and mainly seasonal (gas, hydro dams).

### Electricity import/export

**Fig 10:**
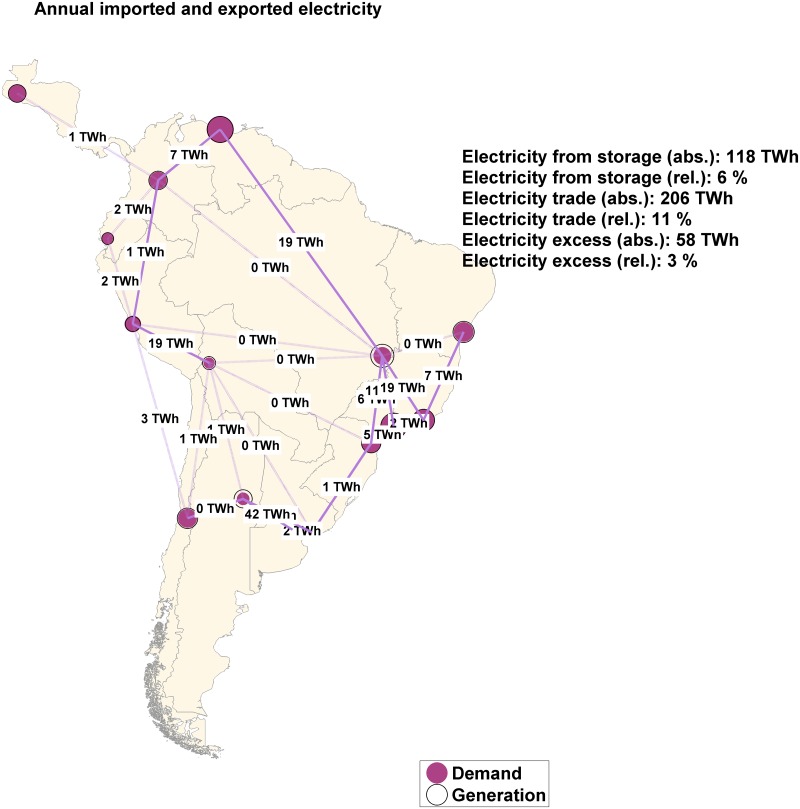
Annual generation and demand diagrams for area-wide open trade scenario.

For the region-wide open trade scenario, all sub-regions of South and Central America need to match their demand using only their own RE resources. Nonetheless, in the case of the country-wide and area-wide open trade scenarios, a division of sub-regions into net exporters and net importers with interregional electricity flows can be observed (Fig 10). Net exporters are sub-regions with the best renewable resources and net importers are sub-regions with moderate ones. Due to export and import, there are differences in generation and demand but in a minor quantity also due to storage losses. For the area-wide integrated scenario (not shown in Fig 10) the differences are mainly due to energy consumption for SNG production.

Fig 10 reveals the net exporter sub-regions: Central South America, Brazil North, Brazil Northeast, Argentina West and Chile. Net importers are Peru, Argentina Northeast, Argentina East and Brazil Southeast. The remaining sub-regions are classified as balancing sub-regions since electricity is both imported and exported during the day and throughout the year. Hourly resolved profiles for regional generation in an importer sub-region (Argentina Northeast), balancing sub-region (Central America) and exporting region (Brazil North) are presented in Fig A, B and C in S2 File, respectively). Considering the integrated scenario, SNG producing regions tend to increase the intra-regional electricity generation to fulfill the increased demand for the desalination and SNG producing sectors what would change the picture remarkably.

The import/export shares in all regions and scenarios are summarized in Table N in S1 File. The share of export is defined as the ratio of net exported electricity to the generated primary electricity of a sub-region and the share of import is defined as the ratio of imported electricity to the electricity demand. The area average is composed of sub-regions’ values weighted by the electricity demand.

**Fig 11:**
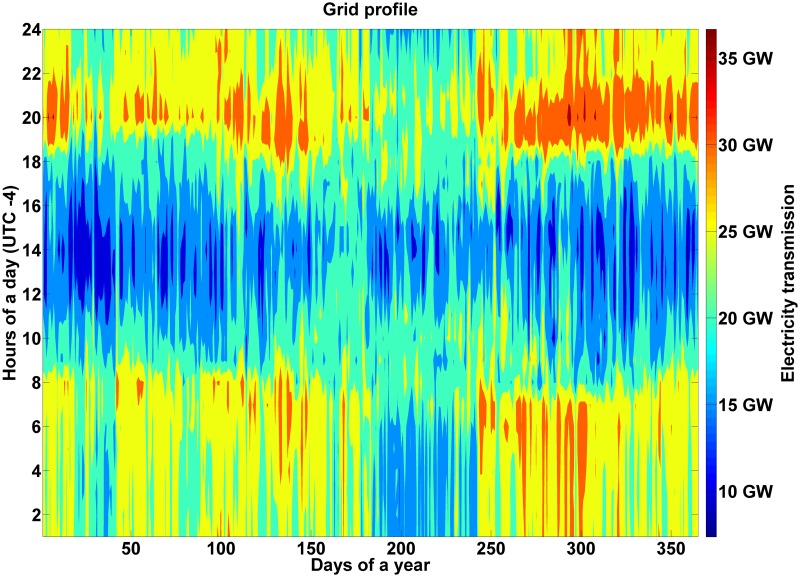
Interregional electricity flows for the area-wide open trade scenario for South and Central America.

Concerning interregional electricity flows between the sub-regions, Fig 11 shows that electricity trade increases during the night and first morning hours all throughout the year and decreases during the same daily period in the winter time. This tendency can be explained by the fact that high shares of solar PV electricity generation requires that during the night, not only storage technologies are used but also electricity is imported by sub-regions with higher inflexible electricity generation. In this case, the electricity flow is directed from sub-regions with high hydropower generation, such as Brazil North and Central South America, to regions with high solar PV generation, such as Venezuela and Peru. During the winter time the electricity demand decreases and, consequently, the need for electricity trade. An overview of the power transmission lines, the key parameters and the percentage of grid utilization can be found in Table O in S1 File.

### Energy flow for 100% RE power systems for South and Central America

**Fig 12:**
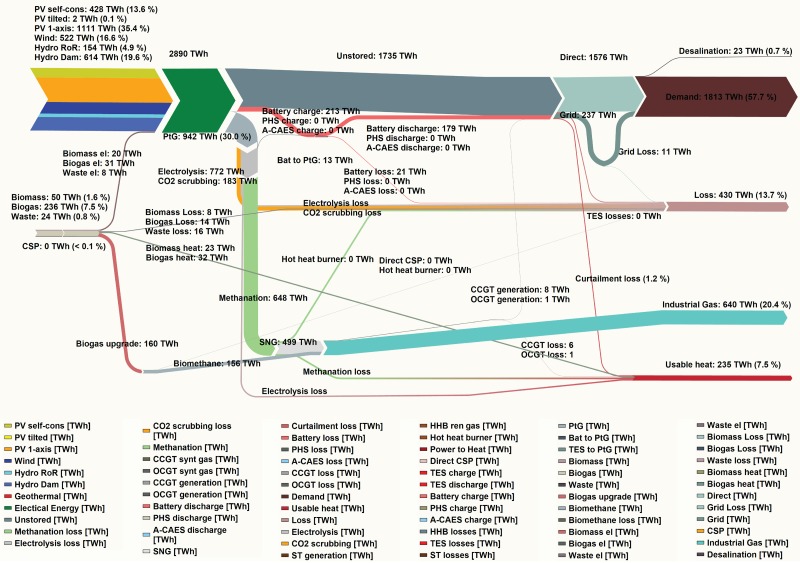
Energy flow of the system for the integrated scenario.

An energy flow diagram is capable of showing the breakdown of energy production, utilization and losses according to each technology and sector. The energy flow for the integrated system is presented in Fig 12; diagrams for the region-wide and area-wide scenarios are presented in Fig F in S2 File.

The flows are comprised of primary RE resource generators, energy storage technologies, HVDC transmission grids, total demand of each sector and system losses. Potentially usable heat and ultimate system losses consist of the difference of primary power generation and final electricity demand. Both are comprised of curtailed electricity; heat produced by biomass, biogas and waste-to-energy power plants; heat of transforming power-to-hydrogen in the electrolyzers, hydrogen-to-methane in methanation and methane-to-power in the gas turbines; and the efficiency losses in A-CAES, PHS, battery storage, as well as by the HVDC transmission grid.

## Discussion

### Power system costs for the studied scenarios

From the presented results for the South and Central America region, and from the results presented in [23] and [27] for the Northeast Asian region, it can be concluded that different levels of grid development lead to different power system configurations and costs. The installation of an HVDC transmission grid between sub-regions enables a significant decrease not only in the cost of electricity but also in RE and storage installed capacities in the RE-based system. The total levelized cost of electricity in the region decreased from 61.9 €/MWh for the region-wide open trade scenario to 59.1 €/MWh for the country-wide open trade scenario and 56.5 €/MWh for the area-wide open trade scenario. The total annualized cost of the system decreased from 115 b€ for the region-wide open trade scenario to 104 b€ for the area-wide open trade scenario. In parallel the capex requirements are reduced from 948 b€ for the region-wide open trade to 912 b€ and 889 b€ for the country-wide and area-wide open trade scenarios, respectively. Additional costs of HVDC transmission lines (56 b€ annual cost for area-wide scenario) are compensated by a substantial decrease in generation and storage capacities enabled by lower losses and costs of energy transmission compared to energy storage, and access to low cost electricity generation in other regions. The HVDC transmission grid may not increase the chances to supply electricity to rural people that do not yet have access to electricity nowadays in South and Central America regions. However, RE-based mini-grid solutions and solar home systems may be a proper solution in addition to grid extension [45,46,47].

### The role and influence of PV technologies on 100% RE system for South and Central America by 2030

PV technologies have the highest share in installed capacities for a 100% RE energy mix in all the analyzed scenarios, which is in accordance with the fact that these technologies have well distributed FLH all over the sub-regions and are the least cost RE technology in most of the cases. Besides, the installation of distributed small-scale and centralized PV plants is already profitable in numerous regions in the word and PV electricity generation cost are set to decrease even more in the coming years [48, 49], especially in regions with high PV FLH.

In addition, PV self-consumption has to be analyzed in more detail since prosumers’ electricity generation provokes some positive and negative distortion in the system demand profile (Fig 5) and costs [23]. In order to measure the influences of PV prosumers, region-wide, country-wide and area-wide open trade scenarios are also calculated without PV self-consumption and the total demand is assumed to be covered by a more centralized system. The annualized costs for the more centralized 100% RE system are 12.2% lower for the region-wide scenario (101 b€ against 115 b€ base scenario), 12.8% lower for the country-wide scenario and 13.5% lower for the area-wide open trade scenario for the RE system without PV self-consumption. This result is explained by the fact that PV self-consumption provokes additional costs because of a different target function of prosumers. Prosumers will install PV systems, if LCOE of PV self-consumption is lower than the grid electricity selling price. However, LCOE of PV self-consumption can be higher than the total system LCOE. Consequently, the system reacts by installing more flexibility granting capacities, such as low cost RE or further storage capacities, which increase the system costs as well., As in South and Central America there are only slight differences in electricity consumption during the whole year, the peak, minimum and average load, and total remaining electricity demand in the system are significantly decreased by 15–23% due to PV prosumers’ electricity production. Thus, the most expensive peak hours throughout the year are substantially reduced by about 15% by PV self-consumption, which exhibits a substantial economic value. The electricity consumption in the centralized system was higher in the first morning hours and during the evenings, and with PV prosumers influence, there was a lower electricity consumption during the afternoon. For the region-wide scenario a comparable low cost increase due to the decentralized generation can be explained by the fact that additional disturbance cost in the system (provoked by prosumers) is compensated by access to low cost residential electricity (for residential consumers WACC is assumed to be 4%). Finally, PV self-consumption is in particular valuable in area constrained regions, since zero impact areas on rooftops can be utilized for local electricity generation, which in turn reduces the requirement of imports. This may be in some regions a policy option for reaching higher local value creation and less supply risk due to higher electricity imports.

### Advantages of the system’s flexibility

The integrated scenario is the scenario in which water desalination and industrial gas sectors are integrated into the power sector. The integration can be considered for the reason that both new integrated sectors require only electricity to cover projected natural gas demand (except the gas demand for power generation and residential purposes that are not considered in this study) and renewable water demand by SNG generation and SWRO desalination, respectively. In parallel with supplying demand, such an integration gives the system additional flexibility, especially for seasonal fluctuation compensation. Variable PtG and desalination plants enable the production of synthetic gas and water during periods of excess electricity, reducing LCOG and LCOW. Recent SWRO desalination plants, for instance, such as the Hadera plant in Israel and Al Khafji in Saudi Arabia have been designed to work on variable power input [35, 50]. Al-Nory and El-Beltagy (2014) also discuss the variable operation of desalination plants depending on the availability of renewable energy in the grid. In 100% RE systems, generation and supply management and grid integration are very important tools that diminish curtailed electricity, integrate other sectors to the power sector and connect RE plants across a wide geographical area complementing their resources. The availability of RE in South and Central America is sufficient to cover additional electricity demand for producing 640 TWh_LHV_ of SNG and 3.9 billion m^3^ of renewable water. Adding 967 TWh_el_ for gas synthesis and SWRO desalination induces an additional installation of RE generation capacities of 410 GW of PV and about 66 GW of wind energy. As well, former long-term gas storage is partly substituted by short-term battery storage. Next, there is a significant increase in electrolyzer units of about 131 GW and substantially reduced gas turbines.

The integration benefit for the electricity, water and industrial gas sectors is estimated to be about 13.1 b€ of the annual system cost. An additional decrease in the electricity demand by 167 TWh and the curtailed electricity by 23 TWh can be observed also. These benefits are of 8%, 5% and 23%, respectively, compared to the non-integrated, separate systems. Further, the cost of renewable water seems to be quite affordable at 0.91 €/m^3^, and the cost of electricity decreases by 18% to 46 €/MWh for the integrated scenario compared to the area-wide open trade scenario without sector integration. However, the cost of synthetic gas, at 95.1 €/MWh_LHV_, appears to be significantly higher than the current price.

### Other alternatives for achieving a low carbon based energy system

The conclusions for this study clearly show the potential of the region for RE generation and for a global climate change mitigation strategy. The results of a fairly low LCOE for the year 2030 (in all the considered scenarios) added to the already existing RE policies and low carbon development plans can boost the development of a renewable power system in the South and Central American region in the coming years. Among the alternatives for achieving a low carbon based energy system, non-renewable options, such as nuclear energy, natural gas and coal carbon capture and storage (CCS) have been also highlighted [51]. The LCOE of the alternatives are as follows [51]: 112 €/MWh for new nuclear (assumed for 2023 in the UK and Czech Republic), 112 €/MWh for gas CCS (assumed for 2019 in the UK) and 126 €/MWh for coal CCS (assumed for 2019 in the UK). However, a report published by [52] concludes that CCS technology is not likely to be commercially available before the year 2030 [23]. In the mid-term, the findings for Europe can be also assumed for South and Central America. These other alternatives have still further disadvantages such as nuclear melt-down risk, nuclear terrorism risk, unsolved nuclear waste disposal, remaining CO_2_ emissions of power plants with CCS technology, a diminishing conventional energy resource base and high health cost due to heavy metal emissions of coal fired power plants. Moreover, the 100% renewable resource-based energy system options for South and Central America presented in this work seem to be considerably lower in cost (about 45–63%) than the other alternatives.

### Comparison to a business as usual scenario

A comparison to a business as usual scenario (BAU) is important in order to check, if the estimated LCOE for the 100% RE system is lower than the LCOE of a system based on current policies. In order to do so, a BAU scenario based on the current policy scenario [1] was analyzed and its LCOE was calculated for the year 2030. The mix of installed capacities for the BAU scenario is displayed in Table 3.

**Table 3:** Total installed capacities for the BAU scenario based on [1].

		BAU scenario
PV	[GW]	11
CSP	[GW]	1
Wind energy	[GW]	32
Biomass power plants	[GW]	22
Gas-fired power plants	[GW]	118
Geothermal energy	[GW]	2
Hydro Run-of-River	[GW]	51
Hydro dams	[GW]	191
Nuclear power plants	[GW]	6
Diesel-fired power plants	[GWh]	16
Oil power plants	[GW]	24
Coal power plants	[GW]	17

Due to the fact that the BAU scenario considers fossil fuel power plants, two different values for LCOE were calculated for this scenario: one that does not take into account CO_2_ emission costs (LCOE_BAU_) and another that considers a 59.8 €/tCO_2_ [53] emission cost (LCOE_BAU-CO2_). An overnight approach was also assumed and transmission costs were not included since AC grids costs and distribution are not available in the literature. Therefore, for comparing LCOE values for BAU and 100% RE scenarios, LCOT was excluded from total LCOE for country and area-wide scenarios.

**Fig 13:**
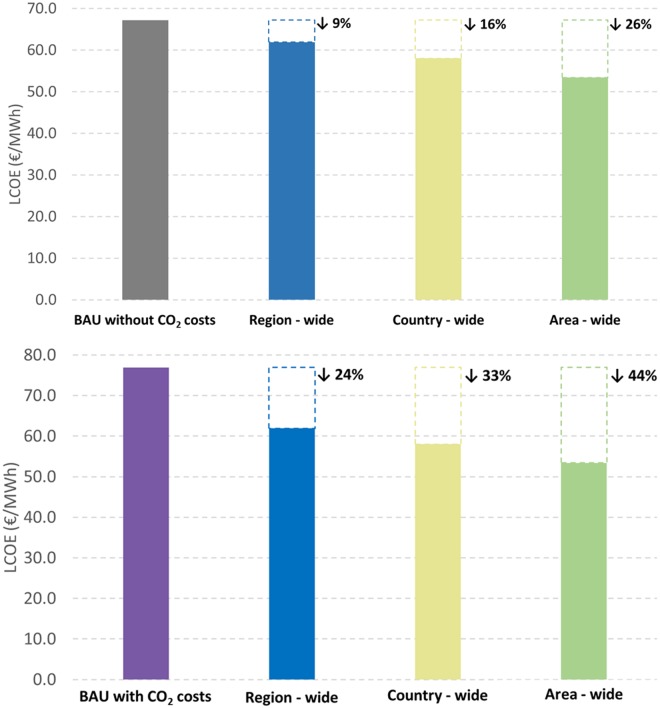
Comparison of LCOE_BAU_, to country-wide, region-wide and area-wide scenarios (top). Comparison of LCOE_BAU-CO2_ to country-wide, region-wide and area-wide scenarios (bottom).

Fig 13 shows the result for LCOE_BAU_ and LCOE_BAU-CO2_ in comparison to LCOE of 100% RE scenarios. The calculated LCOE_BAU_ and LCOE_BAU-CO2_ values are 67.2 €/MWh_el_ and 77.0 €/MWh_el_, respectively. Comparing LCOE_BAU_ to LCOE for 100% RE scenarios, the values are at least 9 and at most 16% lower, what shows that even under no CO_2_ emission taxes policy, a 100% RE power system is the least cost solution for the increase in the region’s electricity demand by 2030. In addition, if CO_2_ emission costs are considered, these percentages are even higher ranging from 24 to 44% as shown on Fig 13. Although the discussion of other costs and benefits (such as decrease of air pollution, increase in health and quality of life, minimal impact on the environment and economic benefits to regional areas) are not the scope of this paper, it is important to mention that if these costs and benefits are included in the calculations, they would increase even more LCOE of BAU scenarios and decrease LCOE of 100% RE scenarios. As pointed out by [54], [55] and [56] these costs are very high and further substantially increase the real societal costs of current conventional energy systems and have to be regarded as societal very harmful subsidies. For this reason, it is essential to reinforce the importance of a new policy scenario and of the development of RE technologies in South and Central America.

### Limitations of the considered model

The model presented in this article presents some limitations that had to be mentioned and analysed. Most of them come from future financial, technical, demographic and economic development assumptions and from technological change and climate policies that were not considered. The key limitations are listed below:

- Since financial and technical assumptions for the renewable energy and storage technologies are global in nature, differences between the assumptions for countries and regions of the same country are not considered. Therefore, divergences in total cost and mix of capacities may be found if a further study that takes into account different assumptions for South and Central American countries is considered.
- Biomass potentials used in this study do not consider differences in biomass availability during the year, i.e. it does not consider that straw and bagasse from sugarcane refineries are available only during the harvest season. This may lead to an overestimation of biomass power plant installed capacities and an underestimation of storage capacities in the current study.
- It was considered that PV prosumers’ surplus electricity production can be fed into the grid for a transfer selling price of 2 €cents/kWh. However, this is a broader estimation in order to calculate the benefits from the selling of excess energy by prosumers and does not consider that each South and Central American country has its own policy (feed-in-tariff or RE auctions) and selling regulation and price.
- Grid interconnections are based on each country’s current national grid although the model’s sub-regional division does not allow that the modelled system accurately represents the current system. In addition, most of the grid interconnection between countries do not yet exist, and were considered in order to show the benefits from grid integration in energy systems with high shares of RE. AC transmission lines were not modelled due to: the lack of information on this data for the entire region; a considerable increase in the computational cost that could unfeasible the model.
- The overnight approach can increase the LCOE of primary generation in regions with already existing high shares of hydropower plants, such as Brazil South. This fact can considerably change the total mix of capacity of the whole region if another approach that considered already existing power plants and future power plants were regarded.

## Conclusion

RE technologies can generate enough energy to cover all electricity demand in South and Central America for the year 2030 on a price level of 47–62 €/MWh_el_, depending on geographical and sectoral integration. The electricity needed to cover PtG technology and SWRO desalination demand can be produced by RE sources as well, providing the region with 100% renewable synthetic natural gas and clean water supply. However, due to high cost obtained for the synthetic gas, government regulation and/or subsidies might be needed to ensure the financial viability of this synthetic fuel, as part of a comprehensive net zero emission strategy.

Due to the need to diminish the dependency of the South and Central American power sector on a changing hydrological profile, different shares of variable RE technologies are essential for 100% RE-based power systems in the region. This need has been an urgent issue for many countries within the region in recent years. In the cost minimized design of the energy mix presented in this study, hydropower continues to dominate in the electricity sector (in terms of TWh of electricity production) in most sub-regions of South and Central America. Nonetheless, the vulnerability of the existing power system is solved by a high share of complementary renewable sources, leading to the least-cost solution for the problem under the given constraints.

For all the studied scenarios solar PV technology emerged as the main energy supply (in terms of GW of installed capacities) in most of the sub-regions; however, with the integration of industrial natural gas and water desalination sectors, the role of PV decreases in sub-regions where wind turbines offer the least cost technology. The HVDC transmission grid plays a key role within the renewable resource-based energy system since the established Super Grid enables a significant cost decrease, a cut-off of storage utilization, and a significant reduction of primary generation capacities. Meanwhile, PV self-consumption induces a moderate increase in total electricity costs of 12–14%. This is due to the fact that consumers tend to utilize higher cost level solar energy and the excess electricity from prosumer generation provokes additional disturbances in the system. In turn, this increases the system need for flexibility.

For the integrated scenario it was found that industrial SNG generation displaces SNG storage as seasonal storage for the electricity sector. Instead of gas turbine utilization in case of an energy deficit, the system curtails the SNG generation in that system set-up as a major source of flexibility to the system.

A fully integrated renewable energy system has to be simulated and deeply studied in order to better understand the findings for the South and Central American region. However, compared to a BAU scenario based on current policies, this research work indicates that a 100% renewable resources-based energy system is a real economic, environmental and health low cost option and is a very important indicator that should be taken into account by policymakers for the development of future policies.

## Supporting information

S1 File Table A: Financial assumptions for energy system components [36, 49, 57, 58, 59, 60, 61, 62,]. Table B: Efficiencies and energy to power ratio of storage technologies. Assumptions are mainly taken from [61]. Table C: Efficiency assumptions for energy system components for the 2030 reference years. Assumptions are mainly taken from [59] and from [61]. Table D: Efficiency assumptions for HVDC transmission [63]. Table E: Regional end-user grid electricity costs for year 2030. Assumptions for most of the countries were taken from [39]. Table F: Average full load hours and LCOE for optimally tilted and single-axis tracking PV systems, and wind power plants in Central and South American regions. Abbreviation: full load hour, *FLH*. Table G: Regional biomass [13] and geothermal energy potentials. Table H: Regional biomass costs, calculated based on biomass sources mix in the region. Solid wastes cost are based on assumption of 75 €/ton gate fee paid to the MSW incinerator. Table I: Lower limits of installed capacities in South and Central American regions. Data were taken from [3]. Table J: Upper limits on installable capacities in South and Central America regions in units of GW_th_ for CSP and GW_el_ for all other technologies. Table K: Annual industrial gas [37, 1] and water demand [36] for year 2030. Table L: Overview on prosumers electricity costs installed capacities and energy utilization for South and Central America.Table M: Overview on storage capacities, throughput and full cycles per year for the four scenarios for South and Central America. Table N: Total LCOE components in all sub-regions. Table O: Overview on electricity transmission lines parameters for the area-wide open trade scenario.
(DOCX)

S2 File Fig A. Hourly generation profile for Argentina Northeast, example for an importing region. Fig B. Hourly generation profile for Central America, example for a balancing region. Fig C. Hourly generation profile for Brazil North, example for an exporting region. Fig D. Aggregated state-of-charge for the storages in the integrated scenario: battery (top left), PHS (top right), A-CAES (bottom left) and gas storage (bottom right). Fig E. State-of-charge for hydro dams in the integrated scenario. During the rainy season, the water levels in the reservoirs are above 60% of the reservoir storage capacities. Fig F. Energy flow of the system for the region-wide open trade (top) and area-wide open trade (bottom) scenarios.
(RAR)

## Abbreviations

BAU: Business as usual scenario
Capex: capital expenditures
CCGT: combined cycle gas turbine
ccs: carbon capture and storage
chp: combined heat and power
csp: concentrating solar thermal power
el: electricity
fix: fixed
gdp: gross domestic product
gt: gas turbine
HHV: base on higher heating value of fuel
hvdc: high voltage direct current
lcoc: levelized cost of curtailment
lcoe: levelized cost of electricity
lcoebau: levelized cost of electricity of BAU scenario
lcoebau-CO2: levelized cost of electricity of BAU scenario considering CO2 costs
lcog: levelized cost of gas
lcos: levelized cost of storage
lcot: levelized cost of transmission
lcow: levelized cost of water
LHV: base on lower heating value of fuel
ocgt: open cycle gas turbine
opex: operational expenditures
phs: pumped hydro storage
PtG: power-to-gas
PV: photovoltaic
RE: renewable energy
RoR: run-of-river
sng: synthetic natural gas
st: steam turbine
swro: seawater reverse osmosis
tes: thermal energy storage
th: thermal
wacc: weighted average cost of capital
