# Supplementary material for: Hydro, wind and solar power as a base for a 100% renewable energy supply for South and Central America
Source: PLoS One. 2017 Mar 22;12(3):e0173820. doi: 10.1371/journal.pone.0173820 (PMC5362076; doi:10.1371/journal.pone.0173820)
Supplement: S1 File — Table A: Financial assumptions for energy system components [36, 49, 57, 58, 59, 60, 61, 62,]. Table B: Efficiencies and energy to power ratio of storage technologies. Assumptions are mainly taken from [61]. Table C: Efficiency assumptions for energy system components for the 2030 reference years. Assumptions are mainly taken from [59] and from [61]. Table D: Efficiency assumptions for HVDC transmission [63]. Table E: Regional end-user grid electricity costs for year 2030. Assumptions for most of the countries were taken from [39]. Table F: Average full load hours and LCOE for optimally tilted and single-axis tracking PV systems, and wind power plants in Central and South American regions. Abbreviation: full load hour, FLH. Table G: Regional biomass [13] and geothermal energy potentials. Table H: Regional biomass costs, calculated based on biomass sources mix in the region. Solid wastes cost are based on assumption of 75 €/ton gate fee paid to the MSW incinerator. Table I: Lower limits of installed capacities in South and Central American regions. Data were taken from [3]. Table J: Upper limits on installable capacities in South and Central America regions in units of GWth for CSP and GWel for all other technologies. Table K: Annual industrial gas [37, 1] and water demand [36] for year 2030. Table L: Overview on prosumers electricity costs installed capacities and energy utilization for South and Central America.Table M: Overview on storage capacities, throughput and full cycles per year for the four scenarios for South and Central America. Table N: Total LCOE components in all sub-regions. Table O: Overview on electricity transmission lines parameters for the area-wide open trade scenario. (DOCX) [file pone.0173820.s001.docx]

**Supporting Information – S1 File**

**Table A: Financial assumptions for energy system components [36, 49, 57, 58, 59, 60, 61, 62]**

| **Technology** | **Capex**  **[€/kW]** | **Opex fix**  **[€/(kW.a)]** | **Opex var**  **[€/(kWh)]** | **Lifetime**  **[a]** |
| --- | --- | --- | --- | --- |
| PV optimally tilted | 550 | 8 | 0 | 35 |
| PV single-axis tracking | 620 | 9 | 0 | 35 |
| PV rooftop | 813 | 12 | 0 | 35 |
| Wind onshore | 1000 | 20 | 0 | 25 |
| CSP (solar field) | 528 | 11 | 0 | 25 |
| Geothermal | 4860 | 87 | 0 | 30 |
| Hydro run-of-river | 2560 | 115.2 | 0.005 | 60 |
| Hydro dam | 1650 | 66 | 0.003 | 60 |
| Water electrolysis | 380 | 13 | 0.0012 | 30 |
| Methanation | 234 | 5 | 0.0015 | 30 |
| CO_2_ scrubbing | 356 | 14 | 0.0013 | 30 |
| CCGT | 775 | 19.4 | 0.001 | 30 |
| OCGT | 475 | 14.25 | 0.001 | 30 |
| Steam turbine | 600 | 12 | 0 | 30 |
| Hot heat burner | 100 | 2 | 0 | 30 |
| Heating rod | 20 | 0.4 | 0.001 | 30 |
| Biomass CHP | 2500 | 175 | 0.001 | 30 |
| Biogas CHP | 370 | 14.8 | 0.001 | 30 |
| Waste incinerator | 5240 | 235.8 | 0.007 | 20 |
| Biogas digester | 680 | 27.2 | 0 | 20 |
| Biogas upgrade | 250 | 20 | 0 | 20 |
|  |  |  |  |  |
|  | **Capex**  **[€/(m^3^∙a)]** | **Opex fix**  **[€/(m^3^∙a)]** | **Opex var**  **[€/m^3^]** | **Lifetime**  **[a]** |
| Water desalination | 2.23 | 0.09 | 0 | 30 |
|  | **Capex**  **[€/(kWh)]** | **Opex fix**  **[€/(kWh.a)]** | **Opex var**  **[€/kWh]** | **Lifetime**  **[a]** |
| Battery | 150 | 10 | 0.0002 | 10 |
| PHS | 70 | 11 | 0.0002 | 50 |
| A-CAES | 31 | 0.4 | 0.0012 | 40 |
| TES | 24 | 2 | 0 | 20 |
| Gas storage | 0.05 | 0.001 | 0 | 50 |
|  |  |  |  |  |
|  | **Capex**  **[€/(m^3^)]** | **Opex fix**  **[€/(m^3^∙h∙a)]** | **Opex var**  **[€/m^3^]** | **Lifetime**  **[a]** |
| Water storage | 65 | 1.3 | 0 | 30 |

|  | **Capex**  **[€/(kW_NTC_∙km)]** | **Opex fix**  **[€/(kW_NTC∙_km∙a)]** | **Opex var**  **[€/kWh_NTC_]** | **Lifetime**  **[a]** |
| --- | --- | --- | --- | --- |
| HVDC line on ground | 0.612 | 0.0075 | 0 | 50 |
| HVDC line submarine | 0.992 | 0.0010 | 0 | 50 |
|  |  |  |  |  |
|  | **Capex**  **[€/(m^3^∙h∙km)]** | **Opex fix**  **[€/(m^3^∙h∙km∙a)]** | **Opex var**  **[€/m^3^∙h∙km]** | **Lifetime**  **[a]** |
| Horizontal pumping and pipes | 19.3 | 0.39 | 0 | 30 |
| Vertical pumping and pipes | 15.5 | 0.31 | 0 | 30 |

**Table B: Efficiencies and energy to power ratio of storage technologies. Assumptions are mainly taken from [61].**

| **Technology** | **Efficiency  [%]** | **Energy/Power Ratio  [h]** | **Self-Discharge  [%/h]** |
| --- | --- | --- | --- |
| Battery | 90 | 6 | 0 |
| TES | 90 | 8 | 0.002 |
| PHS | 85 | 8 | 0 |
| A-CAES | 70 | 100 | 0.001 |
| Gas storage | 100 | 80*24 | 0 |

**Table C: Efficiency assumptions for energy system components for the 2030 reference years. Assumptions are mainly taken from [59] and from [61].**

|  | **η_el_ [%]** | **η_th_ [%]** |
| --- | --- | --- |
| CSP (solar field) |  | 51 |
| Steam turbine | 42 |  |
| Hot heat burner |  | 95 |
| Heating rod |  | 99 |
| Water electrolysis |  | 84 |
| Methanation |  | 77 |
| CO_2_ scrubbing |  | 78 |
| CCGT | 58 |  |
| OCGT | 43 |  |
| Geothermal | 24 |  |
| Biomass CHP | 40 | 45 |
| Biogas CHP | 42 | 43 |
| Waste incinerator | 34 |  |
| Biogas upgrade |  | 98 |

**Table D: Efficiency assumptions for HVDC transmission [63].**

|  | **Power losses** |
| --- | --- |
| HVDC line | 1.6%/1000 km |
| HVDC converter pair | 1.4% |

**Table E: Regional end-user grid electricity costs for year 2030. Assumptions for most of the countries were taken from [39].**

| **Region** | **Electricity costs [€/MWh]** | | |
| --- | --- | --- | --- |
|  | **Residential** | **Commercial** | **Industrial** |
| Total area | 188 | 175 | 159 |
| Central America | 169 | 164 | 159 |
| Colombia | 160 | 164 | 165 |
| Venezuela | 39 | 35 | 46 |
| Ecuador | 127 | 127 | 127 |
| Peru | 193 | 165 | 123 |
| Central South America | 121 | 101 | 84 |
| Brazil South | 250 | 220 | 190 |
| Brazil São Paulo | 250 | 220 | 190 |
| Brazil Southeast | 250 | 220 | 190 |
| Brazil North | 250 | 220 | 190 |
| Brazil Northeast | 250 | 220 | 190 |
| Argentina Northeast | 170 | 140 | 115 |
| Argentina East | 170 | 140 | 115 |
| Argentina West | 170 | 140 | 115 |
| Chile | 215 | 201 | 185 |

**Table F: Average full load hours and LCOE for optimally tilted and single-axis tracking PV systems, and wind power plants in Central and South American regions. Abbreviation: full load hour, *FLH*.**

| **Region** |  | **Pop. [mio. Pop]** | **Electr. demand [TWh]** | **PV fixed tilted**  **FLH** | **PV single-axis**  **FLH** | **Wind  FLH** | **PV fixed tilted  LCOE [€/MWh]** | **PV single-axis  LCOE [€/MWh]** | **Wind**  **LCOE [€/MWh]** |
| --- | --- | --- | --- | --- | --- | --- | --- | --- | --- |
| Total area |  | 526 | 1813 | 1800 | 2031 | 3066 | 28 | 28 | 34 |
| Central America |  | 55 | 105 | 1633 | 2141 | 1557 | 31 | 27 | 68 |
| Colombia |  | 53 | 108 | 1520 | 1835 | 799 | 33 | 31 | 132 |
| Venezuela |  | 38 | 252 | 1573 | 1981 | 980 | 32 | 29 | 108 |
| Ecuador |  | 20 | 52 | 1510 | 1942 | 1939 | 34 | 29 | 55 |
| Peru |  | 37 | 98 | 1820 | 2414 | 1815 | 28 | 24 | 58 |
| Central South America |  | 21 | 43 | 1775 | 2341 | 2813 | 29 | 24 | 38 |
| Brazil South |  | 33 | 141 | 1470 | 1877 | 2012 | 34 | 30 | 53 |
| Brazil São Paulo |  | 50 | 240 | 1544 | 1984 | 1653 | 33 | 29 | 64 |
| Brazil Southeast |  | 46 | 183 | 1588 | 2069 | 1541 | 32 | 28 | 69 |
| Brazil North |  | 36 | 111 | 1499 | 1904 | 823 | 34 | 30 | 129 |
| Brazil Northeast |  | 63 | 141 | 1668 | 2296 | 3371 | 30 | 25 | 31 |
| Argentina Northeast |  | 14 | 55 | 1497 | 1957 | 2877 | 34 | 18 | 37 |
| Argentina East |  | 23 | 96 | 1532 | 2008 | 3824 | 33 | 28 | 28 |
| Argentina West |  | 17 | 59 | 1799 | 2425 | 4801 | 28 | 24 | 22 |
| Chile |  | 20 | 130 | 1909 | 2640 | 4513 | 27 | 22 | 23 |

**Table G: Regional biomass [13] and geothermal energy potentials.**

| **Region** | **Biomass potential [TWh_LHV_/a]** | | | **Geothermal** |
| --- | --- | --- | --- | --- |
|  | **Solid waste** | **Solid biomass** | **Biogas sources** | **Potentials [TWh_th_/a]** |
| Total area | 23.6 | 765.2 | 236.1 | 255.0 |
| Central America | 4.4 | 17.9 | 15.5 | 0 |
| Colombia | 1.7 | 15.5 | 15.4 | 0 |
| Venezuela | 1.7 | 11.7 | 7.3 | 0 |
| Ecuador | 1.2 | 5.2 | 3.7 | 42.0 |
| Peru | 0.9 | 7.7 | 4.8 | 0 |
| Central South America | 1.4 | 17.0 | 5.1 | 0 |
| Brazil South | 0.7 | 57.7 | 24.7 | 0 |
| Brazil São Paulo | 1.1 | 72.5 | 37.4 | 0 |
| Brazil Southeast | 1.1 | 78.3 | 34.9 | 54.2 |
| Brazil North | 0.8 | 180.0 | 27.5 | 0 |
| Brazil Northeast | 1.4 | 122.3 | 47.8 | 0 |
| Argentina Northeast | 0.7 | 30.3 | 2.3 | 0 |
| Argentina East | 1.2 | 57.7 | 4.6 | 0 |
| Argentina West | 0.9 | 48.5 | 3.3 | 31.3 |
| Chile | 4.4 | 42.9 | 1.8 | 127.5 |

**Table H: Regional biomass costs, calculated based on biomass sources mix in the region. Solid wastes cost are based on assumption of 75 €/ton gate fee paid to the MSW incinerator.**

| **Region** | **Biomass costs [€/MWh_th_]** | | |
| --- | --- | --- | --- |
|  | **Solid waste** | **Solid biomass** | **Biogas sources** |
| Total area | -15.05 | 8.88 | 9.58 |
| Central America | -15.10 | 5.34 | 10.20 |
| Colombia | -15.25 | 5.34 | 9.52 |
| Venezuela | -15.16 | 5.52 | 7.11 |
| Ecuador | -14.84 | 5.32 | 7.40 |
| Peru | -15.25 | 5.30 | 6.59 |
| Central South America | -14.90 | 5.26 | 7.15 |
| Brazil South | -15.25 | 8.08 | 10.60 |
| Brazil São Paulo | -15.25 | 6.30 | 10.60 |
| Brazil Southeast | -15.25 | 7.71 | 10.60 |
| Brazil North | -15.25 | 13.57 | 10.60 |
| Brazil Northeast | -15.25 | 8.81 | 10.60 |
| Argentina Northeast | -15.06 | 5.95 | 7.72 |
| Argentina East | -15.01 | 5.48 | 8.61 |
| Argentina West | -15.01 | 6.96 | 8.61 |
| Chile | -14.69 | 15.02 | 0 |

**Table I: Lower limits of installed capacities in South and Central American regions. Data were taken from [3].**

| **Region** | **Installed capacity [MW]** | | | |
| --- | --- | --- | --- | --- |
|  | **Solar PV** | **Wind** | **Hydro**  **RoR and dams** | **PHS** |
| Total area | 1160.5 | 9371.8 | 163310 | 1100 |
| Central America | 42.5 | 470 | 5490 | 0 |
| Colombia | 17.6 | 19.5 | 11120 | 0 |
| Venezuela | 59.2 | 101 | 20940 | 0 |
| Ecuador | 14.5 | 88.9 | 2320 | 0 |
| Peru | 100 | 146.8 | 3740 | 0 |
| Central South America | 8.5 | 54 | 8990 | 0 |
| Brazil South | 3 | 1068 | 23720 | 0 |
| Brazil São Paulo | 1.1 | 0 | 13890 | 0 |
| Brazil Southeast | 3 | 29.2 | 15040 | 126 |
| Brazil North | 100.4 | 680.7 | 27230 | 0 |
| Brazil Northeast | 51 | 4571 | 12080 | 0 |
| Argentina Northeast | 6 | 311.7 | 5120 | 0 |
| Argentina East | 32.7 | 98.9 | 0 | 0 |
| Argentina West | 5 | 413.1 | 7250 | 974 |
| Chile | 716 | 1319 | 6380 | 0 |

**Table J: Upper limits on installable capacities in South and Central America regions in units of GW_th_ for CSP and GW_el_ for all other technologies.**

| **Region** | **area** |  | **Limits [GW]** | | | | | | | | | |
| --- | --- | --- | --- | --- | --- | --- | --- | --- | --- | --- | --- | --- |
|  | **[1000 km^2^]** | **Solar**  **CSP** | **Solar**  **PV** | **Wind** | **Hydro RoR** | **Hydro dams** | | | **PHS** | | |  |
| Total area | 18313 | 247247 | 82415 | 6153 | 57 | 188 | | 2.2 | | | |  |
| Central America | 522 | 7051 | 2350 | 175 | 4 | 4 | | 0 | | |  |  |
| Colombia | 1142 | 15414 | 5138 | 384 | 4 | 12 | | 0 | | |  |  |
| Venezuela | 1374 | 18554 | 6185 | 462 | 20 | 12 | | 0 | | |  |  |
| Ecuador | 256 | 3461 | 1154 | 86 | 1 | 2 | | 0 | | |  |  |
| Peru | 1285 | 17350 | 5783 | 432 | 3 | 3 | | 0 | | |  |  |
| Central South America | 1505 | 20322 | 6774 | 506 | 1 | 12 | | 0 | | |  |  |
| Brazil South | 577 | 7786 | 2595 | 194 | 3 | 32 | | 0 | | |  |  |
| Brazil São Paulo | 248 | 3351 | 1117 | 83 | 3 | 18 | | 0 | | |  |  |
| Brazil Southeast | 676 | 9131 | 3044 | 227 | 4 | 19 | | 0.2 | | |  |  |
| Brazil North | 5460 | 73711 | 24570 | 1835 | suppl7 | 34 | | 0 | | |  |  |
| Brazil Northeast | 1554 | 20983 | 6994 | 522 | 1 | 17 | | 0 | | |  |  |
| Argentina Northeast | 678 | 9149 | 3050 | 228 | 2 | 6 | | 0 | | |  |  |
| Argentina East | 308 | 4155 | 1385 | 103 | 0 | 0 | | 0 | | |  |  |
| Argentina West | 1971 | 26610 | 8870 | 662 | 0 | 11 | | 2 | | |  |  |
| Chile | 757 | 10219 | 3406 | 254 | 4 | 6 | | 0 | | |  |  |
|  |  |  |  |  |  | |  | | |  | | |

**Table K: Annual industrial gas [37, 41] and water demand [36] for year 2030.**

| **Region** | **Annual gas demand** | **Annual electricity demand for gas synthesis** | **Annual water desalination demand** | | | **Annual electricity demand for water desalination** |
| --- | --- | --- | --- | --- | --- | --- |
|  | **TWh_th_** | **TWh_el_** | | **10^6^ m^3^** | **TWh_el_** | |
| Total area | 640.0 | 943.9 | | 3935.2 | 23.12 | |
| Central America | 3.2 | 0 | | 0 | 0 | |
| Colombia | 36.4 | 49.3 | | 0 | 0 | |
| Venezuela | 228.6 | 427.7 | | 244.6 | 5.9 | |
| Ecuador | 1.0 | 0 | | 0 | 0 | |
| Peru | 13.9 | 20.3 | | 3.3 | 0.01 | |
| Central South America | 12.1 | 16.9 | | 0 | 0 | |
| Brazil South | 55.2 | 73.3 | | 0 | 0 | |
| Brazil São Paulo | 72.9 | 90.4 | | 0 | 0 | |
| Brazil Southeast | 41.7 | 33.9 | | 0 | 0 | |
| Brazil North | 20.3 | 2.8 | | 0 | 0 | |
| Brazil Northeast | 26.8 | 0 | | 8.7 | 0.01 | |
| Argentina Northeast | 23.7 | 42.2 | | 0 | 0 | |
| Argentina East | 53.6 | 96.6 | | 0 | 0 | |
| Argentina West | 39.1 | 70.4 | | 1095.9 | 2.9 | |
| Chile | 11.5 | 20.1 | | 2582.7 | 14.3 | |

**Table L: Overview on prosumers electricity costs installed capacities and energy utilization for South and Central America.**

| Prosumers parameters | **Residential** | **Commercial** | **Industrial** |
| --- | --- | --- | --- |
| Electricity price [€/kWh] | 0.188 | 0.175 | 0.159 |
| PV LCOE [€/kWh] | 0.053 | 0.081 | 0.081 |
| Self-consumption PV LCOE [€/kWh] | 0.080 | 0.105 | 0.104 |
| Self-consumption PV and Battery LCOE [€/kWh] | 0.083 | 0.111 | 0.109 |
| Self-consumption LCOE [€/kWh] | 0.073 | 0.104 | 0.103 |
| Benefit [€/kWh] | 0.115 | 0.070 | 0.056 |
| Installed capacities | **Residential** | **Commercial** | **Industrial** |
| PV [GW] | 155.5 | 54.4 | 107.9 |
| Battery storage [GWh] | 148.1 | 91.9 | 172.0 |
| Generation | **Residential** | **Commercial** | **Industrial** |
| PV [TWh] | 167.5 | 86.6 | 174.3 |
| Battery storage [TWh] | 44.7 | 28.8 | 58.9 |
| Excess [TWh] | 56.3 | 20.3 | 38.8 |
| Utilization | **Residential** | **Commercial** | **Industrial** |
| Self-consumption of generated PV electricity [%] | 66.4 | 76.6 | 77.7 |
| Self-coverage market segment [%] | 19.9 | 18.3 | 18.4 |
| Self-coverage operators [%] | 99.3 | 91.6 | 92.0 |

**Table M: Overview on storage capacities, throughput and full cycles per year for the four scenarios for South and Central America.**

|  |  | | **Region-wide** | **Country-wide** | **Area-wide** | **Integrated** |
| --- | --- | --- | --- | --- | --- | --- |
|  | Battery SC | [GWh_el_] | 412 | 412 | 412 | 412 |
| Storage capacities | Battery system | [GWh_el_] | 250 | 242 | 126 | 195 |
|  | PHS | [GWh_el_] | 1.2 | 1.1 | 1.1 | 1.1 |
|  | A-CAES | [GWh_el_] | 122.6 | 0.2 | 8.7 | 0 |
|  | Gas | [GWh_th_] | 73559 | 78999 | 63832 | 42723 |
|  | Battery SC | [TWh_el_] | 132.4 | 132.4 | 132.4 | 132.4 |
| Throughput of storages | Battery system | [TWh_el_] | 81.5 | 78.7 | 40.4 | 59.6 |
|  | PHS | [TWh_el_] | 0.1 | 0.1 | 0.1 | 0.2 |
|  | A-CAES | [TWh_el_] | 3.3 | 0 | 0.2 | 0 |
|  | Gas | [TWh_th_] | 155.6 | 158.5 | 133.8 | 15.6 |
|  | Battery SC | [-] | 321.4 | 321.4 | 321.4 | 321.4 |
| Full cycles per year | Battery system | [-] | 326.5 | 325.1 | 320.7 | 305.3 |
|  | PHS | [-] | 113.7 | 100.7 | 126.6 | 185.8 |
|  | A-CAES | [-] | 27.2 | 27.6 | 24.5 | 24.2 |
|  | Gas | [-] | 2.1 | 2.0 | 2.1 | 0.4 |

**Table N: Total LCOE components in all sub-regions.**

| **Region-wide** | **LCOE primary** | **LCOC** | **LCOS** | **LCOT** | **LCOE total** |  |
| --- | --- | --- | --- | --- | --- | --- |
|  | **[€/MWh]** | **[€/MWh]** | **[€/MWh]** | **[€/MWh]** | **[€/MWh]** |  |
| Area average | 41.6 | 3.0 | 17.3 | 0 | 61.9 |  |
| Central America | 41.1 | 2.0 | 16.7 | 0 | 59.8 |  |
| Colombia | 42.2 | 1.4 | 11.0 | 0 | 54.6 |  |
| Venezuela | 45.2 | 1.5 | 33.3 | 0 | 80.0 |  |
| Ecuador | 40.0 | 1.1 | 15.3 | 0 | 56.4 |  |
| Peru | 38.5 | 0.8 | 17.7 | 0 | 57.0 |  |
| Central South America | 41.9 | 10.7 | 13.1 | 0 | 65.7 |  |
| Brazil South | 49.9 | 3.5 | 12.0 | 0 | 65.4 |  |
| Brazil São Paulo | 38.6 | 1.4 | 12.3 | 0 | 52.3 |  |
| Brazil Southeast | 44.7 | 2.4 | 16.4 | 0 | 63.5 |  |
| Brazil North | 44.1 | 12.7 | 25.3 | 0 | 82.1 |  |
| Brazil Northeast | 38.4 | 2.6 | 12.0 | 0 | 53.0 |  |
| Argentina Northeast | 46.1 | 4.2 | 11.7 | 0 | 62.0 |  |
| Argentina East | 39.0 | 2.1 | 19.7 | 0 | 60.8 |  |
| Argentina West | 35.9 | 5.4 | 6.2 | 0 | 47.5 |  |
| Chile | 33.0 | 2.5 | 15.5 | 0 | 51.0 |  |
|  |  |  |  |  |  |  |
| **Country-wide** | **LCOE primary** | **LCOC** | **LCOS** | **LCOT** | **LCOE total** | **export (-)/ import (+)** |
|  | **[€/MWh]** | **[€/MWh]** | **[€/MWh]** | **[€/MWh]** | **[€/MWh]** | **[%]** |
| Area average | 40.2 | 2.2 | 15.6 | 1.1 | 59.1 | 8.1 |
| Central America | 41.1 | 2.0 | 16.7 | 0 | 59.8 | 0 |
| Colombia | 42.2 | 1.3 | 11.1 | 0 | 54.6 | 0 |
| Venezuela | 45.2 | 1.5 | 33.3 | 0 | 80.0 | 0 |
| Ecuador | 40.0 | 1.1 | 15.3 | 0 | 56.4 | 0 |
| Peru | 38.5 | 0.8 | 17.7 | 0 | 57.0 | 0 |
| Central South America | 41.9 | 10.7 | 13.1 | 0 | 65.7 | 0 |
| Brazil South | 51.1 | 0.3 | 12.4 | 1.6 | 65.4 | 1.5 |
| Brazil São Paulo | 35.6 | 0.3 | 12.9 | 0.8 | 49.6 | 6.5 |
| Brazil Southeast | 40.9 | 1.3 | 15.1 | 1.8 | 59.1 | 14.3 |
| Brazil North | 40.7 | 8.0 | 10.1 | 2.4 | 61.2 | -23.9 |
| Brazil Northeast | 38.7 | 2.7 | 11.2 | 0.2 | 52.8 | -1.4 |
| Argentina Northeast | 52.3 | 1.8 | 7.8 | 2.8 | 64.7 | 17.8 |
| Argentina East | 33.8 | 1.7 | 13.6 | 8.8 | 57.9 | 48.3 |
| Argentina West | 30.1 | 2.5 | 4.4 | 3.7 | 40.7 | -45.3 |
| Chile | 33.0 | 2.5 | 15.5 | 0 | 51.0 | 0 |
| **Area-wide** | **LCOE primary** | **LCOC** | **LCOS** | **LCOT** | **LCOE total** | **export (-)/ import (+)** |
|  | **[€/MWh]** | **[€/MWh]** | **[€/MWh]** | **[€/MWh]** | **[€/MWh]** | **[%]** |
| Area average | 40.6 | 1.3 | 11.5 | 3.1 | 56.5 | 13.6 |
| Central America | 40.8 | 1.5 | 16.8 | 0.5 | 59.6 | 0.7 |
| Colombia | 44.8 | 0.2 | 10.6 | 4.3 | 59.9 | -8.1 |
| Venezuela | 49.0 | 0.6 | 14.7 | 3.1 | 67.4 | 9.1 |
| Ecuador | 40.8 | 0.2 | 10.4 | 1.6 | 53.0 | 7.0 |
| Peru | 32.5 | 0.3 | 13.7 | 3.6 | 50.1 | 18.7 |
| Central South America | 43.2 | 0.8 | 3.6 | 5.1 | 52.7 | -28.0 |
| Brazil South | 52.4 | 0.1 | 13.6 | 3.7 | 69.8 | 7.8 |
| Brazil São Paulo | 35.2 | 0.2 | 12.6 | 1.3 | 49.3 | 3.2 |
| Brazil Southeast | 40.3 | 1.1 | 14.8 | 2.8 | 59.0 | 12.1 |
| Brazil North | 40.6 | 4.9 | 9.6 | 4.6 | 59.7 | -29.4 |
| Brazil Northeast | 38.6 | 2.7 | 10.6 | 0.7 | 52.6 | -4.4 |
| Argentina Northeast | 49.1 | 0.7 | 6.4 | 3.9 | 60.1 | 14.9 |
| Argentina East | 33.3 | 1.4 | 8.9 | 10.1 | 53.7 | 39.8 |
| Argentina West | 31.2 | 1.5 | 3.5 | 5.7 | 41.9 | -44.1 |
| Chile | 33.9 | 1.6 | 10.9 | 1.2 | 47.6 | -3.3 |
| **Integrated scenario** | **LCOE primary** | **LCOC** | **LCOS** | **LCOT** | **LCOE total** | **export (-)/ import (+)** |
|  | **[€/MWh]** | **[€/MWh]** | **[€/MWh]** | **[€/MWh]** | **[€/MWh]** | **[%]** |
| Area average | 36.3 | 1.0 | 7.6 | 1.7 | 46.6 | 9.1 |
| Central America | 41.0 | 1.5 | 16.5 | 0.6 | 59.6 | 1.8 |
| Colombia | 40.4 | 0.7 | 6.0 | 1.9 | 49.0 | -4.4 |
| Venezuela | 35.3 | 1.1 | 8.5 | 0.5 | 45.4 | 1.0 |
| Ecuador | 39.3 | 0.3 | 11.9 | 1.3 | 52.8 | 5.6 |
| Peru | 30.2 | 0.3 | 11.6 | 2.3 | 44.4 | 12.1 |
| Central South America | 41.8 | 0.5 | 2.2 | 3.0 | 47.5 | -15.4 |
| Brazil South | 45.0 | 1.4 | 6.9 | 1.9 | 55.2 | 7.5 |
| Brazil São Paulo | 33.9 | 0.6 | 7.5 | 1.4 | 43.4 | 5.6 |
| Brazil Southeast | 39.6 | 0.5 | 10.0 | 3.3 | 53.4 | 17.5 |
| Brazil North | 42.0 | 2.1 | 6.6 | 4.7 | 55.4 | -28.1 |
| Brazil Northeast | 37.0 | 1.6 | 8.9 | 1.6 | 49.1 | -11.9 |
| Argentina Northeast | 42.9 | 0.7 | 3.2 | 2.9 | 49.7 | 17.0 |
| Argentina East | 29.6 | 0.8 | 3.3 | 2.0 | 35.7 | 13.0 |
| Argentina West | 27.7 | 0.8 | 1.9 | 2.7 | 33.1 | -25.0 |
| Chile | 31.4 | 0.9 | 8.1 | 0.9 | 41.3 | -6.4 |

Table O: Overview on electricity transmission lines parameters for the area-wide open trade scenario.

|  | **Region 1** | **Region 2** | **Length** | **Capacity** | **Utilisation** |
| --- | --- | --- | --- | --- | --- |
|  |  |  | **[km]** | **[GW]** | **[%]** |
| 1 | Central America | Colombia | 2319 | 0.6 | 83 |
| 2 | Colombia | Venezuela | 1131 | 6.9 | 65 |
| 3 | Colombia | Ecuador | 1090 | 1.0 | 70 |
| 4 | Colombia | Peru | 2054 | 1.0 | 81 |
| 5 | Venezuela | Brazil North | 3912 | 4.3 | 71 |
| 6 | Ecuador | Peru | 1252 | 0.9 | 74 |
| 7 | Peru | Central South America | 1183 | 4.6 | 57 |
| 8 | Peru | Chile | 2709 | 0.8 | 79 |
| 9 | Central South America | Argentina West | 1894 | 0.7 | 78 |
| 10 | Central South America | Chile | 2089 | 0.7 | 77 |
| 11 | Brazil South | Brazil São Paulo | 368 | 5.7 | 61 |
| 12 | Brazil South | Brazil North | 1195 | 1.1 | 87 |
| 13 | Brazil South | Argentina Northeast | 1358 | 1.8 | 71 |
| 14 | Brazil São Paulo | Brazil Southeast | 398 | 2.8 | 71 |
| 15 | Brazil São Paulo | Brazil North | 970 | 1.9 | 82 |
| 16 | Brazil Southeast | Brazil North | 1039 | 3.2 | 81 |
| 17 | Brazil Southeast | Brazil Northeast | 1322 | 1.3 | 76 |
| 18 | Argentina Northeast | Argentina East | 448 | 2.1 | 59 |
| 19 | Argentina Northeast | Argentina West | 924 | 1.5 | 65 |
| 20 | Argentina East | Argentina West | 703 | 6.8 | 71 |
| 21 | Argentina West | Chile | 717 | 1.2 | 61 |
